# Fungal Biorefinery: Mushrooming Opportunities

**DOI:** 10.1002/gch2.202400315

**Published:** 2024-11-21

**Authors:** Mitchell P. Jones, Alexander Bismarck

**Affiliations:** ^1^ Institute of Material Chemistry and Research, Polymer and Composite Engineering (PaCE) Group, Faculty of Chemistry University of Vienna Währinger Straße 42 Vienna 1090 Austria

Fungal biorefinery is a research area that has evolved very rapidly over the past decade. Historically, materials research relating to fungi has addressed topics with largely negative implications for society: most often relating to wood decay that would invariably compromise structures and infrastructure. Over the past decade, we have witnessed and contributed to an unexpected rise in fungal‐derived materials research that instead capitalizes on the positive characteristics of fungi to create novel materials and processes. We are pleased to present this special issue on fungal biorefinery, showcasing some of the most recent research in a field that just a couple of decades ago would, in the words of most mycologists, have been considered unlikely at best.

**Figure 1:**
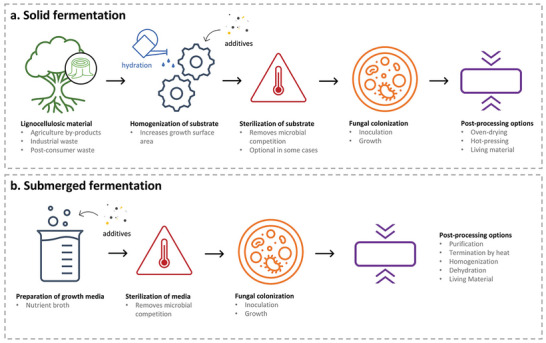
a) Solid and b) (submerged) liquid‐state fermentation processes for the production of fungal materials. Reproduced from 2300140.

Fungal biorefinery contributes practical solutions to many *Global Challenges* objectives. Filamentous fungal growth (hyphae) can be used to bind agricultural and forestry residue together in a natural heterotrophic process that has captured the imagination of academics and industry alike (**Figure** **1**). This process is now used to create foam‐like materials for packaging and thermal and acoustic insulation for non‐structural and semi‐structural applications by an ever‐increasing number of start‐up companies and academics across the globe. While the scalability of these materials remains to be proven, their material properties do for the most part meet the requirements for such applications. These materials provide opportunities for waste upcycling, bio‐based manufacturing and (depending on the process variables) may represent low‐energy manufacturing, carbon sequestration, and generation of biodegradable materials that are highly relevant to sustainability, climate change, and environmental protection goals.

Fungal (nano)materials, typically produced as sheets of micro‐ to nanoscale (hyphae) fibrils in the form of as‐grown or engineered, e.g., through papermaking processes, networks also capitalize on the mechanical properties of chitin‐β‐glucan complex present in hyphal cell walls and can be tuned to exhibit high‐strength or toughness. Conversion of fungal chitin to chitosan lends ion chelation and biomedical properties to the network, which endows them with potential applications in water safety and healthcare. Notable hydrophobic surface properties (although not affecting moisture sorption) exhibited by some fungal nanomaterials coupled with tuneable thermal decomposition and electrical conductivities achieved through biomineralization within hyphal structures add to the versatility of these materials and enable applications in energy storage.

The scope achieved by this special issue is broad: It provides perspective on the influence of genetics, nutrition, and environmental conditions on the properties of fungal materials (2300140, 2300197). Although limited by inherent genetic constraints, the quantity and properties of fungal mycelium are greatly influenced by nutrient type and availability and their growth environment. The interaction between fungi and their growth environment also provides opportunities for biomonitoring and the creation of living composite systems (2400104). Fungi utilize biochemical and electrical signaling pathways to respond to external stimuli within their dynamic growth environment. Understanding the role of hyphal structures in signaling, relevant pathways, and current electrophysiological metrology are key to the creation of new advanced materials.

Original research papers also describe the valorization of food waste, in this case bread, to create novel tuneable fungal monofilaments (2300098). Protonation of the amino groups of cell wall chitosan, followed by homogenization and concentration enabled the spinning of a hydrogel with tensile strengths of up to 140 MPa. Glycerol post‐treatments also enabled elongation at breaks of up to 14%. These properties were comparable to other natural fibers with the monofilaments also biocompatible with human cells and hint at the potential use of fungal monofilaments in textile applications.

Building on a self‐healing mechanism that dates back to Roman times, fungi can promote calcium carbonate precipitation to “heal” cracks in concrete (2300160). Biomineralization of *Trichoderma reesei* in liquid media containing calcium lactate provided greater biomass production but fewer crystals compared to calcium chloride. Nutrient supplementation using potato dextrose broth also increased biomass generated and calcium carbonate precipitated. This has potential applications in the self‐healing of structural construction materials, such as concrete.

Fungal hyphae templates after carbonization are mesoporous and microscale with a size regime close to carbon fibers and BET surface areas of 60–282 m^2^ g^−1^, which greatly exceed values associated with carbon fibers and non‐activated pyrolyzed bacterial cellulose and are approximately on par with values for carbon black and carbon nanotubes (2300315). They also exhibit a higher specific capacitance than some traditional carbon sources, which coupled with the potential to tune their properties through species‐ and growth‐environment‐associated differences in network and filament morphology and inclusion of inorganic material through biomineralization, makes them potentially useful in creating supercapacitors.

Fungal biorefinery is a manufacturing platform with diverse waste input stream options, e.g., food, agricultural, forestry residues, fermentation routes, e.g., agitated or static (surface) liquid state or solid‐state fermentation, and output products, e.g., composite foams for packaging, absorption, thermal or acoustic insulation or fungal nanomaterials for dressings, scaffolds, paper, membranes, yarns and textiles, and coatings. This platform represents flexible and circular bio‐based manufacturing options with the potential to produce many essential products currently reliant on fossil‐derived polymers. The full potential of the fungal biorefinery as a tool in our transition towards a sustainable future is yet to be realized but important areas of future research will include scalability, improvement of life cycle parameters, and improving material properties to ensure that these novel materials can meet the property envelopes required for the applications that they are intended to service.

